# Von Hippel-Lindau gene single nucleotide polymorphism (rs1642742) may be related to the occurrence and metastasis of HBV-related hepatocellular carcinoma

**DOI:** 10.1097/MD.0000000000027187

**Published:** 2021-09-03

**Authors:** Xuebing Chen, Hao Zhang, Shimei Ou, Huijuan Chen

**Affiliations:** Department of Infectious Diseases, the People's Hospital of Deyang City, Sichuan Province, China.

**Keywords:** hepatitis B viruses, hepatocellular carcinoma, rs1642742, single nucleotide polymorphism, Von Hippel-Lindau

## Abstract

It is well-known that microRNAs are able to regulate the expression of target mRNAs through complementary base-pairing to their 3′-untranslated regions (3′UTR) sequences. This study aimed to investigate whether single nucleotide polymorphisms resided in the 3′UTR sequences in patients with chronic hepatitis B viruses (HBV) infection are associated with the development and metastasis of hepatocellular carcinoma (HCC). Seventeen single nucleotide polymorphisms in the 3′UTR sequence of 10 genes regulated or affected by hepatitis B virus X protein were found by bioinformatics methods. Two hundred fifteen patients with HBV-related HCC and 216 patients with chronic HBV infection were recruited. Through case-control study, only found that the von Hippel-Lindau gene rs1642742 (G>A) may be associated with the occurrence and metastasis of HCC. The ORs of the frequencies of rs1642742 A allele versus G allele were 1.424 (*P* = .038, 95% confidence interval [CI] = 1.019–1.989) between HBV-related HCC and chronic HBV infection group and were 2.004 (*P* = .037, 95%CI = 1.031–3.895) between tumor metastasis and non-metastasis group, respectively. Through multivariate regression analysis, we also found that rs1642742 AA genotype was an independent risk factor for tumor metastasis (odds ratio = 2.227, 95% CI = 1.043–4.752, *P* = .038) in HBV-related HCC group. Our study suggested that Von Hippel-Lindau rs1642742 contributed to susceptibility to developing HCC and correlated with tumor metastasis.

## 1 Introduction

Hepatitis B virus-related hepatocellular carcinoma (HBV-related HCC), which is caused by chronic infection of HBV, is one of the most common cancers and a serious threat to public health worldwide.^[1]^ Although chronic HBV infection causes more than 50% of HCC cases worldwide^[2]^ and is the dominant risk factor for HCC in China, only about 14.2% of patients chronically infected with HBV suffer from HCC in their lifetime.^[3]^ This indicates that the occurrence of HBV-related HCC is affected by several factors including both viral and host factor and then many studies have been conducted to screen out risk factors for HBV-related HCC. Until recently, viral factors, including HBV genotypes and mutations, have been identified to affect the risk of HBV-related HCC.^[4–6]^ Hepatitis B virus X protein (HBx protein), another viral factor, is found out to play a vital role in the development of HBV-related HCC.^[7]^ Mounting evidences reveal that HBx is able induce HCC through activating NF-kappa B signaling pathway, JAK-STAT signaling pathway, PI3K-Akt signaling pathway, and MAPK signaling pathway.^[7]^ These signaling pathway genes including Transcription factor p65, Proto-oncogene c-Rel, NFKB inhibitor alpha, Janus kinase, Signal transducer and activator of transcription, Protein inhibitor of activated STAT, Phosphatidylinositol 4,5-bisphosphate 3-kinase catalytic subunit, Protein kinase, Matrix metallopeptidase, and so on. More than that, HBx could also promote carcinogenesis through activating the expression of many important genes including TNF-α, CA9, c-Jun, and so on.^[7,8]^

Meanwhile, growing evidences demonstrate that host genetic background also contributes to the risk of HBV-related HCC.^[9,10]^ MicroRNAs (miRNAs) are 18 to 25 nucleotides (nt), single-stranded non-coding small RNAs.^[11]^ It is well-known that miRNAs are able to regulate the expression of target mRNAs through complementary base-pairing to their 3′-untranslated regions (3′UTR) sequences.^[12]^ Single nucleotide polymorphisms (SNPs) are the most abundant genetic markers in human genome.^[13]^ Because sequence complementarity and binding thermodynamics is important for the interaction between miRNAs and their targets,^[14]^ SNPs in the miRNA-binding sites may disrupt the interaction between miRNAs and their target mRNAs, modulate the expression of these genes and influence the risk of several diseases including non-small cell lung cancer,^[15]^ autism,^[16]^ breast cancer,^[17]^ Burkitt lymphoma,^[18]^ esophageal squamous cell carcinoma,^[19]^ and so on. Recent experimental evidences show that several SNPs in the miRNA binding sites, such as rs1292037,^[20]^ rs2596542,^[21]^ rs1057317,^[22]^ and rs7963551,^[23]^ elevate the risk of HCC.

Because of the importance of above-mentioned signaling pathways genes regulated by HBx, we speculate that SNPs in miRNA-binding sites, which binding in the 3′UTR sequences of these genes, may lead to aberrant gene expression and influence the risk of occurrence HBV-related HCC. To date, there are few studies that have investigated the association between the SNPs in miRNA-binding sites binding with the genes regulated or affected by HBx and HBV-related HCC. In this study, we tried to investigate whether these SNPs could increase or decrease the risk of occurrence HBV-related HCC. With the use of bioinformatic tools, the putative functional SNPs in the miRNAs binding sites were identified and selected for this study. Through case-control studies, we found that SNP in miRNA-binding sites maybe related to the occurrence and metastasis of HBV-related HCC for the first time. This study provides new insights into the developing and progression of HBV-related HCC in viral and host factors.

## 2 Methods and materials

### 2.1 Ethics approval

All clinical work was conducted in accordance with the ethical regulations of World Medical Association Declaration of Helsinki (1996) after the approval of the ethical committee of Faculty of the People's Hospital of Deyang City, Sichuan Province, China. All methods were performed in accordance with the relevant guidelines and regulations. Patients or legal guardians received detailed information about the study, and they signed an informed consent form.

### 2.2 Study population

Between September 2016 and December 2018, we consecutively enrolled HBV-related HCC patients who were admitted to People's Hospital of Deyang City. Patients with chronic HBV infection who matched the gender and age of HBV-related HCC patients were also recruited. The detailed inclusion and exclusion criteria in the current study were as follows: clinical diagnosed HCC patients; hepatitis B surface antigen positive for more than 6 months; negative test results for hepatitis A, C, D, and E viruses, cytomegalovirus, Epstein-Barr virus, and HIV; no decompensated cirrhosis, liver failure; and no alcoholic hepatitis, autoimmune hepatitis, and fatty liver. All subjects were ethnic Han Chinese. HCC and chronic HBV infection were diagnosed according to “Diagnosis, Staging, and Management of Hepatocellular Carcinoma: 2018 Practice Guidance by the American Association for the Study of Liver Diseases”^[24]^ and “The guideline of prevention and treatment for chronic hepatitis B: a 2015 update”.^[25]^ Informed consents were obtained from all the patients involved in this study. Clinical information (including HBV-DNA and Hepatitis B e antigen [HBeAg] serostatus) were gathered from all patients.

### 2.3 Genetic screening

The PubMed database was thoroughly searched for relevant studies published from 1990 to December 2018 with the following keywords “hepatitis B virus X protein”, “HBx”, “signaling pathway”, “Hepatocellular Carcinoma”, and “HCC”. Ninety-two genes interacting with HBx or involving in signal pathways transactivated by HBx had been screened and included in this study (Table S1, Supplemental Digital Content).

### 2.4 SNP selection

The 3′UTR sequences of these genes were obtained from UCSC database (http://genome.ucsc.edu). All SNPs in the 3′UTR with a minor allele frequency higher than 0.1 in Chinese population were identified through an extensive search in dbSNP database (https://www.ncbi.nlm.nih.gov/snp). Using the tools provided by the website (http://www.microrna.org/microrna/home.do) to identify SNPs at putative miRNA binding sites. The influence of these SNPs on the interaction between miRNAs and their targets were evaluated through calculating the change of minimum free energy (MFE, expressed in KJ/mol) between the 2 alleles through the online software-RNAhybrid (http://bibiserv.techfak.uni-bielefeld.de/rnahybrid/).^[26]^ As proposed by Nicoloso et al,^[27]^ SNPs causing MFE changed more than 8% were considered biologically relevant and included in the study. As a result, 17 SNPs in 10 genes were selected for genotyping (Table 1).

**Table 1:** The detailed information of SNPs in putative miRNA binding sites.

Gene	SNP ID	miRNA	MFE (KJ/mol)	MFE change (%)
AKT2	rs33933140^∗^	miR-216a	1.5	7.7
		miR-216b	1.6	9.5
		miR-490-3p	2.0	8.0
AKT3	rs9428966	miR-135a	0.7	3.7
APC	rs3733961^∗^	miR-487b	1.8	12.5
BIRC5	rs2239680^∗^	miR-335	4.1	27.7
	rs1042489^∗^	miR-708	0.7	3.7
		miR-28-5p	0.0	0.0
		miR-132	1.0	5.0
		miR-211-3p	1.7	–9.1
		miR-7113-5p	3.2	–17.8
		miR-505-5p	3.9	–21.5
	rs1042541^∗^	miR-138	0.8	3.3
		miR-125a-3p	1.2	5.1
		miR-133a-5p	0.0	0.0
		miR-138-5p	0.4	–1.6
		miR-6131	–1.9	9.0
	rs1042542^∗^	miR-4325	4.7	–27.0
		miR-7703	4.7	–22.5
CTNNB1	rs2953	miR-296-3p	–1.8	–7.7
GRB2	rs7219^∗^	miR-326	–2.8	–13.6
		miR-330-5p	0.6	2.5
JAK3	rs3008	miR-1308	1.7	–8.7
KRAS	rs712^∗^	miR-422a	–1.6	–6.4
		miR-378	–2.1	–8.5
		miR-193b	1.4	7.3
		miR-200b	0.3	1.9
		miR-200c	0.0	0.0
		miR-429	0.0	0.0
	rs9266^∗^	miR-181d	1.5	10.4
		miR-181b	–1.1	–6.7
		miR-181c	0.1	0.6
		miR-181a	0.0	0.0
	rs13096	miR-101	–1.3	–7.7
	rs1137188^∗^	miR-129-5p	–1.5	–8.6
		miR-421	0.5	3.0
MAPK1	rs13515	miR-187	0.9	4.9
	rs3810610	miR-34c-5p	0.0	0.0
		miR-210	1.8	7.8
MAPK10	rs958^∗^	miR-125a-5p	4.5	30.6
		mir-125b	6.5	42.8
		miR-431	–0.9	–4.2
		miR-4319	6.0	–34.9
		miR-125b-5p	6.5	–31.0
		miR-125a-5p	4.9	–26.1
MET	rs41738	miR-139-5p	0.0	0.0
MYD88	rs7744	miR-218	–1.2	–6.9
		miR-376a	0.0	0.0
		miR-376b	0.0	0.0
NFKBIA	rs8904^∗^	miR-450a	1.3	13.4
	rs696^∗^	miR-4692	0.8	–3.0
		miR-4514	1.6	–7.7
		miR-4673	4.9	–21.2
		miR-4645-5p	5.3	–29.3
		miR-4775-3p	6.5	–28.9
		miR-4640-5p	6.5	–18.3
		miR-4726-5p	5.9	–19.4
		miR-6508-3p	6.3	–26.9
		miR-6724-5p	3.6	–11.8
		miR-6773-5p	4.1	–15.2
	rs2273650^∗^	miR-4459	1.1	–5.8
		miR-4700-3p	4.3	–22.5
		miR-7151-3p	5.3	–26.2
		miR-5095	3.9	–20.4
PIK3CG	rs3173908^∗^	miR-539	1.7	10.8
PIK3R3	rs1707337	miR-875-5p	–0.6	–3.0
SPP1	rs1126772	miR-23a	–1.2	–6.7
		miR-23b	0.1	0.6
		miR-371-5p	0.1	0.7
STAM	rs2764805^∗^	miR-199a-5p	–1.8	8.3
		miR-199b-5p	–3.6	20.1
REL	rs3732179	miR-29a-3p	2.9	–14.6
		miR-29b-3p	2.9	–15.1
		miR-29c-3p	0.0	0.0
VEGFA	rs10434	miR-140-5p	–0.5	–2.1
VHL	rs1642742a	miR-381	–0.2	–1.0
		miR-300	–4.6	–25.7

MFE = minimum free energy, miRNA = microRNA, SNP = single nucleotide polymorphism.

∗ These SNPs were included in our case-control study.

### 2.5 DNA extraction and SNP genotyping

**Figure 1:**
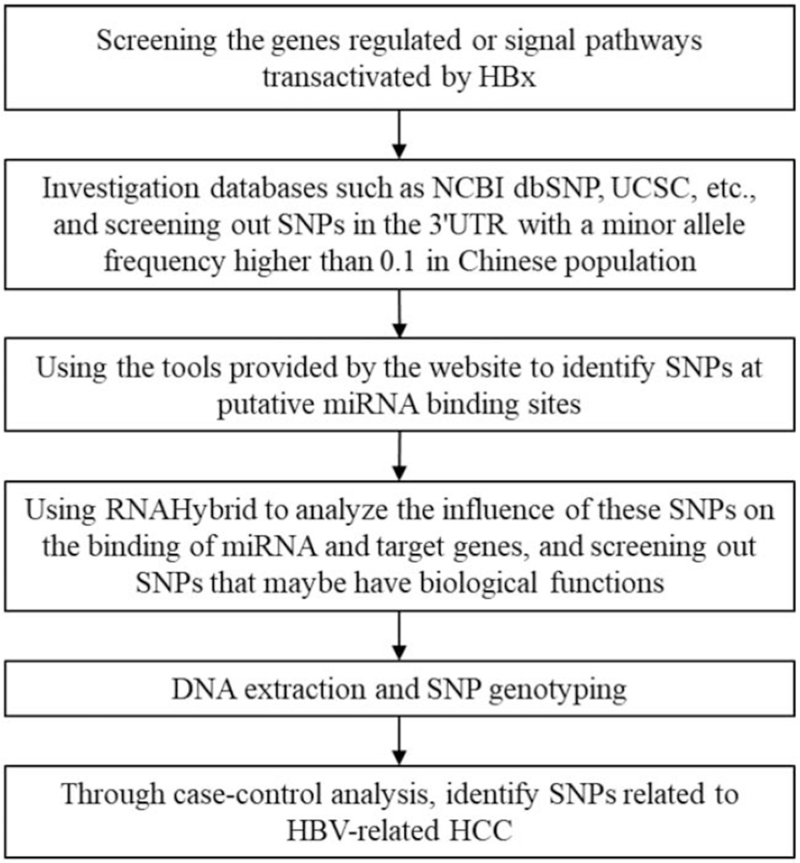
Flowchart of experimental research.

The genomic DNA was isolated from blood clot using TIANamp Blood Clot DNA Kit (Tiangen biotech Co. Ltd, Beijing, China) according to manufacturer's protocol and stored at –80°C for genotyping. All samples were genotyped using matrix-assisted laser desorption ionization-time of-flight mass spectrometry (Sequenom Inc., San Diego, CA). Amplification primers and extension primers were designed using theAssayDesigner3.1 software (Sequenom Inc). Polymerase chain reaction (PCR) amplification of target sequence was performed in a multiplex reaction containing 1 μL genomic DNA. After PCR amplification, remaining dNTPs were dephosphorylated by shrimp alkaline phosphatase (Sequenom Inc.). Then, extension primers were used for locus-specific single-base extensions. The extension products were purified by cation-exchange resin (Sequenom Inc.), transferred onto the 384-well spectroCHIP (Sequenom Inc.) and genotyped using a matrix-assisted laser desorption ionization-time of-flight mass spectrometer. Genotyping data were analyzed using the MassARRAYTyper software version 3.4 (Sequenom Inc.). The full research processes are shown in Figure 1.

### 2.6 Statistical analysis

Allele and genotype frequencies were calculated by counting for each locus. The deviation of these SNPs from the Hardy–Weinberg equilibrium was evaluated by Chi-square tests. Continuous variables were expressed as means ± standard deviation and compared by *t* test. Categorical variables were assessed by Chi-square test. Logistic regression was performed to analyze the association between SNPs and HBV-related HCC using gender, age, HBV DNA, and HBeAg serostatus as covariates. Biological and clinical variables were compared between HBV-related HCC group and chronic HBV infection group by Student *t* test and Chi-square test for continuous and categorical variables, respectively. Statistical analysis was performed using the SPSS statistical software package version 19.0 (IBM Corp, Armonk, NY).

## 3 Results

### 3.1 Patient characteristics

In this study, 215 patients with HBV-related HCC and 216 patients with chronic HBV infection were recruited and assigned to HBV-related HCC group and chronic HBV infection group, respectively. Detailed characteristics of patients involved in this study are shown in Table 2. Serum albumin and cholinesterase representing liver synthesis were significantly lower in HBV-related HCC group than in the chronic HBV infection group (*P* < .01). Similarly, the proportion of patients with cirrhosis is significantly higher in HBV-related HCC than the group of chronic HBV infection (36.74% vs 15.74%, *P* < .001). The proportion of patients treated with nucleoside analogues was 14.88% in HBV-related HCC group, which was significantly lower than the patients in chronic HBV infection group (*P* < .001). The level of alpha-fetoprotein in HCC group was higher than the chronic HBV infection group (*P* < .001). The proportion of males, HBeAg positive, and drinking was not significantly different between HBV-related HCC group and chronic HBV infection group (Table 2).

**Table 2:** Clinical characteristics of the patients at baseline.

Characteristics	HBV-related HCC group (n = 215)	Chronic HBV infection group (n = 216)	t (χ^2^)	*P* value
Males, n (%)	190 (88.37)	183 (84.72)	1.233	.267
Age, yrs	53.50 ± 10.97	54.19 ± 9.45	–0.697	.486
HBeAg (+)	33 (15.35)	21 (9.72)	3.113	.078
HBV-DNA <10^5^ copies/mL, n (%)	138 (64.19)	185 (85.65)	26.429	<.001
Total bilirubin, μmol/L	25.84 ± 54.23	24.87 ± 46.04	0.183	.855
Direct bilirubin, μmol/L	17.65 ± 45.00	15.41 ± 29.61	0.538	.591
ALT, U/L	62.28 ± 95.61	60.55 ± 98.10	0.168	.867
AST, U/L	52.56 ± 72.36	50.77 ± 70.02	0.237	.813
Albumin, g/L	36.64 ± 6.82	45.09 ± 5.57	–12.599	<.001
Creatinine, μmol/L	75.17 ± 48.66	73.17 ± 29.49	0.374	.708
alpha-fetoprotein, ng/ml	497.14 ± 458.98	13.31 ± 82.16	12.918	<.001
Cholinesterase, U/L	4421.92 ± 2326.50	5822.26 ± 2581.35	–3.412	.001
Drinking (%)	80 (37.21)	63 (29.17)	3.144	.076
Liver cirrhosis (%)	79 (36.74)	34 (15.74)	24.572	<.001
Antiviral with NAs	32 (14.88)	67 (31.02)	15.853	<.001

ALT = alanine aminotransferase, AST = aspartate aminotransferase, HBeAg = hepatitis B e antigen, HBV = hepatitis B virus, HCC = hepatocellular carcinoma, NAs = nucleos(t)ide analogues.

### 3.2 Seventeen SNPs in 10 genes may influence the interaction between miRNAs binding with SNP sites and their targets

Out of 92 genes involved in this study, bioinformatics analysis identified 20 genes with 29 SNPs in the putative miRNA-binding sites. The detailed information of these 29 SNPs such as nucleotide variation, predicted binding miRNAs, MFE (expressed in KJ/mol) for each allele, and the change of MFE were shown in the Table 1. As listed in Table 1, 17 SNPs causing MFE changed more than 8% were selected for genotyping, another 12 SNPs were excluded. In order to evaluate whether this strategy was reasonable to choose functional SNPs, we searched electronic databases including PubMed, MEDLINE, ScienceDirect, Wiley, Web of Science, and Baidu Scholar for functional researches about all of these 29 SNPs. Out of the 17 SNPs included in genotyping, rs1042489,^[28]^ rs2239680,^[29]^ rs696,^[30]^ rs712,^[31]^ and rs9266^[31]^ were confirmed to be functional in previous researches. Meanwhile, none of the 12 SNPs excluded from genotyping were found to be functional. Therefore, bioinformatics analysis used in the present research was a reliable approach to identify functional SNPs.

### 3.3 Frequency and distribution of SNPs in HBV-related HCC group and chronic HBV infection group

The detailed allele frequencies and genotype distributions of these 17 SNPs in HBV-related HCC group and chronic HBV infection group were listed in Table 3. All of these SNPs were in Hardy–Weinberg equilibrium. Only found that rs1642742 (G>A) was associated with the occurrence of HBV-related HCC. The frequency of rs1642742 A allele in Von Hippel-Lindau (*VHL*) was significantly higher in HBV-related HCC group than in chronic HBV infection group (*P* = .038, odds ratio [OR] = 1.424, 95% confidence interval [CI] = 1.019–1.989). In co-dominant model, logistic regression revealed that heterozygotes of rs1642742 in *VHL* were less likely to be HCC than wild-type homozygotes. In dominant model, logistic regression revealed that patients with AA genotype versus AG and GG genotypes had an OR of 1.530 (*P* = .037, 95% CI = 1.025–2.286) for HBV-related HCC. Taken together, these results suggested that rs1642742 might increase the risk of HBV-related HCC and allele A might be a risk factor (Table 3).

**Table 3:** The detailed allele frequencies and genotype distributions in 17 SNPs of patients.

Genotype and allele	HBV-related HCC group (n = 215)	Chronic HBV infection group (n = 216)	*P* value
rs1042489			
TT	75 (37.69)	64 (29.91)	
CT	92 (46.23)	120 (56.07)	
CC	32 (16.08)	30 (14.02)	.129
T allele	242 (60.80)	248 (57.94)	
C allele	156 (39.20)	180 (42.06)	.403
(TT+CT) vs CC			.558
TT vs (CT+CC)			.094
rs1042541			
GG	81 (41.54)	89 (41.59)	
AG	86 (44.10)	105 (49.07)	
AA	28 (14.36)	20 (9.35)	.256
G allele	248 (63.59)	283 (66.12)	
A allele	142 (36.41)	145 (33.88)	.449
(GG+AG) vs AA			.116
GG vs (AG+AA)			.992
rs1042542			
CC	83 (42.35)	89 (41.59)	
CT	83 (42.35)	104 (48.60)	
TT	30 (15.31)	21 (9.81)	.185
C allele	249 (63.52)	282 (65.89)	
T allele	143 (36.48)	146 (34.11)	.478
(CC+CT) vs TT			.092
CC vs (CT+TT)			.877
rs1137188			
AA	116 (59.49)	135 (63.08)	
AG	70 (35.90)	70 (32.71)	
GG	9 (4.62)	9 (4.21)	.757
A allele	302 (77.44)	340 (79.44)	
G allele	88 (22.56)	88 (20.56)	.486
(AA+AG) vs GG			.840
AA vs (AG+GG)			.456
rs1642742			
AA	133 (67.17)	123 (57.21)	
AG	56 (28.28)	78 (36.28)	
GG	9 (4.55)	14 (6.51)	.111
A allele	322 (81.31)	324 (75.35)	
G allele	74 (18.69)	106 (24.65)	.038
(AA+AG) vs GG			.384
AA vs (AG+GG)			.037
rs2239680			
TT	100 (51.28)	120 (55.81)	
CT	81 (41.54)	79 (36.74)	
CC	14 (7.18)	16 (7.44)	.606
T allele	281 (72.05)	319 (74.19)	
C allele	109 (27.95)	111 (25.81)	.491
(TT+CT) vs CC			.919
TT vs (CT+CC)			.358
rs2273650			
CC	113 (56.22)	106 (49.30)	
CT	72 (35.82)	99 (46.05)	
TT	16 (7.96)	10 (4.65)	.067
C allele	298 (74.13)	311 (72.33)	
T allele	104 (25.87)	119 (27.67)	.557
(CC+CT) vs TT			.164
CC vs (CT+TT)			.158
rs2764805			
AA	66 (33.17)	66 (30.99)	
AC	91 (45.73)	106 (49.77)	
CC	42 (21.11)	41 (19.25)	.712
A allele	223 (56.03)	238 (55.87)	
C allele	175 (43.97)	188 (44.13)	.963
(AA+AC) vs CC			.639
AA vs (AC+CC)			.636
rs3173908			
CC	88 (44.44)	107 (50.71)	
CT	88 (44.44)	89 (42.18)	
TT	22 (11.11)	15 (7.11)	.250
C allele	264 (66.67)	303 (71.80)	
T allele	132 (33.33)	119 (28.20)	.112
(CC+CT) vs TT			.159
CC vs (CT+TT)			.205
rs33933140			
GG	52 (26.00)	67 (31.31)	
AG	102 (51.00)	102 (47.66)	
AA	46 (23.00)	45 (21.03)	.489
G allele	206 (51.50)	236 (55.14)	
A allele	194 (48.50)	192 (44.86)	.294
(GG+AG) vs AA			.628
GG vs (AG+AA)			.233
rs3733961			
CC	130 (67.01)	139 (64.65)	
CT	58 (29.90)	69 (32.09)	
TT	6 (3.09)	7 (3.26)	.881
C allele	318 (81.96)	347 (80.70)	
T allele	70 (18.04)	83 (19.30)	.644
(CC+CT) vs TT			.925
CC vs (CT+TT)			.616
rs696			
CC	66 (33.50)	69 (32.55)	
CT	91 (46.19)	102 (48.11)	
TT	40 (20.30)	41 (19.34)	.925
C allele	223 (56.60)	240 (56.60)	
T allele	171 (43.40)	184 (43.40)	.999
(CC+CT) vs TT			.807
CC vs (CT+TT)			.837
rs712			
CC	118 (60.82)	135 (64.29)	
AC	68 (35.05)	66 (31.43)	
AA	8 (4.12)	9 (4.29)	.741
C allele	304 (78.35)	336 (80.00)	
A allele	84 (21.65)	84 (20.00)	.564
(CC+AC) vs AA			.935
CC vs (AC+AA)			.473
rs7219			
TT	125 (64.10)	148 (69.16)	
CT	59 (30.26)	60 (28.04)	
CC	11 (5.64)	6 (2.80)	.281
T allele	309 (79.23)	356 (83.18)	
C allele	81 (20.77)	72 (16.82)	.148
(TT+CT) vs CC			.151
TT vs (CT+CC)			.278
rs8904			
GG	65 (36.11)	69 (33.50)	
AG	81 (45.00)	97 (47.09)	
AA	34 (18.89)	40 (19.42)	.863
G allele	211 (58.61)	235 (57.04)	
A allele	149 (41.39)	177 (42.96)	.659
(GG+AG) vs AA			.895
GG vs (AG + AA)			.590
rs9266			
GG	116 (59.79)	135 (63.08)	
AG	70 (36.08)	70 (32.71)	
AA	8 (4.12))	9 (4.21)	.772
G allele	302 (77.84)	340 (79.44)	
A allele	86 (22.16)	88 (20.56)	.576
(GG+AG) vs AA			.967
GG vs (AG+AA)			.495
rs958			
CC	124 (62.94)	127 (59.07)	
CT	61 (30.96)	80 (37.21)	
TT	12 (6.09)	8 (3.72)	.271
C allele	309 (78.43)	334 (77.67)	
T allele	85 (21.57)	96 (22.33)	.795
(CC+CT) vs TT			.263
CC vs (CT+TT)			.421

HBV = hepatitis B virus, HCC = hepatocellular carcinoma, SNP = single nucleotide polymorphism.

### 3.4 Frequency and distribution of rs1642742 allele in HBV-related HCC patients

In the HCC group, some patients had tumor metastasis, while others did not. We further analyzed the genotype frequency and distribution of the 2 groups of patients with and without metastasis. Fifty-six patients (26.05%) with HBV-related HCC were found to have intra-hepatic or distant metastases, and 159 patients (73.95%) had not metastases. The frequency of rs1642742 A allele in *VHL* was significantly higher in metastasis group than in non-metastasis group (*P* = .037, OR = 2.004, 95% CI = 1.031–3.895). In dominant model, logistic regression revealed that patients with AA genotype versus AG and GG genotypes had an OR of 2.111 (*P* = .047, 95% CI = 1.001–4.456) for metastasis group. While the rs1642742 of *VHL* in co-dominant model and recessive model, logistic regression revealed that there were no differences between metastasis group and without metastasis group (*P* > .05). In summary, these results suggested that rs1642742 might be correlated with the risk of metastasis of HBV-related HCC as well, and AA genotype might be increased the risk of metastasis of HBV-related HCC (Table 4).

**Table 4:** The detailed allele frequencies and genotype distributions of metastasis and non-metastasis patients.

Genotype and allele	Metastasis group (n = 56)	Non-metastasis group (n = 159)	*P* value
rs1642742			
AA	40 (78.43)	93 (63.27)	
AG	10 (19.61)	46 (31.29)	
GG	1 (1.96)	8 (5.44)	.127
A allele	90 (88.24)	232 (78.91)	
G allele	12 (11.76)	62 (21.09)	.037
(AA+AG) vs GG			.304
AA vs (AG+GG)			.047

### 3.5 Regression analysis

**Figure 2:**
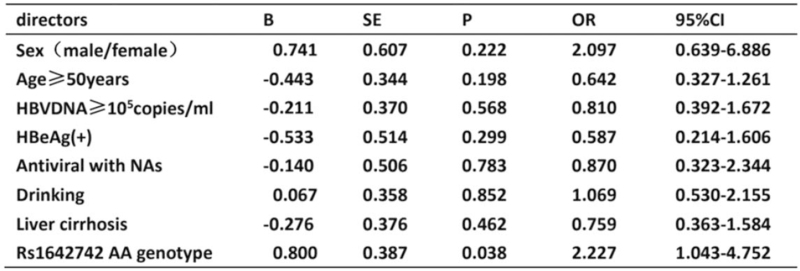
Regression analysis of the risk factors for metastasis of HBV-related HCC. HBV = hepatitis B virus, HCC = hepatocellular carcinoma.

The risk factors for metastasis of HBV-related HCC conferred by the combinations of age, gender, HBeAg, HBV-DNA, cirrhosis, alcohol consumption, antiviral with nucleoside analogues, and rs1642742 AA genotype were assessed using multivariate regression analysis. The results showed that the AA genotype of rs1642742 in HBV-related HCC patients was the only factor related with the cancer metastasis (*P* = .038, OR = 2.227, 95% CI = 1.043–4.752). There was no significant correlation between the other factors including the age more than 50 years, male, HBeAg positive, HBV DNA ≥10^5^ copies/mL, cirrhosis and alcohol consumption, and the metastasis of HCC (Fig. 2).

## 4 Discussion

During recent years, bioinformatics analysis was widely-used to detect SNPs that could interfere with the interaction between miRNAs and their targets.^[27]^ Consistent with previous researches, our study manifested that bioinformatics analysis was a reliable way to identify putative functional SNPs in miRNAs binding sites.

Among 17 SNPs included in our case-control study, rs1042489,^[32]^ rs2273650,^[33]^ rs696,^[33,34]^ and rs712^[35]^ were involved in previous case-control studies. Consistent with previously reports, rs1042489, rs2273650, and rs696 were not associated with the risk of HCC in this study. However, rs712, which was found to increase the risk of HCC by Xiong et al,^[35]^ was not associated with the risk of HCC in our study. A possible reason for this discrepancy might be that the cohort of this study was all chronic HBV infected patients, HBV infection was one of the most important risk factors for HCC as well-known,^[36]^ was not included in Xiong et al's analyses. Although it is still difficult to give a better explanation, further research is needed to confirm whether there is a correlation between HBV infection and rs712 polymorphism.

Association analysis indicated that rs1642742 might be associated with the risk of HBV-related HCC. Further stratification suggested that the allele G of rs1642742 might be a protective allele for patients while the allele A might be associated with tumorigenesis. According to our bioinformatics analysis, the variant allele of rs1642742, which is residing in the 3′UTR of the *VHL* gene, associated with a variety of benign and malignant tumors, may attenuate the expression of *VHL* via strengthen its binding with miRNAs. Mutation of *VHL* gene is associated with several types of tumors like clear-cell renal cancer, pancreatic cancer, pheochromocytoma,^[37–39]^ which suggests that *VHL* gene may play a role as a tumor suppressor in those cancers. Previous researches elucidated that *VHL* was able to form a ternary complex with elongin C and elongin B and induce proteasomal degradation of hypoxia-inducible factors.^[40]^ Moreover, recent study suggested that *VHL* negatively regulated antiviral signaling and affected innate antiviral immunity.^[41]^ Therefore, abnormal expression level of *VHL* caused by rs1642742 may contribute to the risk of HBV-related HCC.

Our results suggested that there was correlation between rs1642742 and metastasis and the allele A might be associated with tumor metastasis. Of course, the correlation between SNP and tumor metastasis had been reported in previous literatures, including lung cancer, breast cancer, gastric cancer, prostate cancer, and so on.^[42–45]^ Rs1642742 is residing in the 3′UTR of the *VHL*, which plays an important role in mRNA translocation, stability and translational regulation. The variant allele of rs1642742 may affect the ability of miRNA to bind to its sequence, which in turn affects the *VHL* gene expression and may lead to tumor metastasis. More researches should be conducted to further evaluate the effect of rs1642742 on the risk of HBV-related HCC and metastasis and clarify its possible mechanism.

Chronic HBV infection can cause chronic hepatitis, cirrhosis, liver failure, and HCC. Liver cirrhosis is an independent risk factor for HCC,^[46]^ which is obtained in this study as well. The high serum HBV-DNA level is closely related to the occurrence of HBV-related HCC, and reducing the viral-load can effectively lead to a decrease in the occurrence of HCC.^[47,48]^ In this study, the proportion of HCC patients with serum HBV-DNA levels <1 × 10^5^ copies/mL was significantly lower than that of patients with chronic HBV infection, which was consistent with the above studies. Long-term antiviral can delay the progression of the disease and reduce the incidence of serious endpoints, such as liver cirrhosis and HCC.^[49]^ Our research also suggests that antiviral HBV therapy may reduce the incidence of HCC.

There are several limitations to our study. First of all, the sample size of our study is relatively small. Multicenter studies with a larger sample size are needed to further validate our findings. Secondly, the functional influence of these SNPs was predicted by bioinformatics analysis in our study. Thus, functional researches should be done to verify their function. Finally, besides SNPs in miRNAs binding sites, SNPs in the promoter region also influence the expression of a gene.^[50]^ Future researches investigating SNPs in the 3′UTR as well as SNPs in the 5′UTR will help us to better understand the mechanism of HBV-related HCC.

In conclusion, through bioinformatics analysis and case-control studies, we found that rs1642742 may be related to the occurrence and metastasis of HBV-related HCC for the first time. Although these results still need to be further validated, our findings provide clues for exploring the mechanism of HBV-related HCC.

## Author contributions

(i) Xuebing Chen is acting as the submission's guarantor (takes responsibility for the integrity of the work as a whole, from inception to published article).

(ii) Author contributions: Study concept and design: Xuebing Chen, Hao Zhang. Analysis and interpretation of data: Xuebing Chen, Hao Zhang, Shimei Ou. Drafting of the manuscript: Xuebing Chen, Hao Zhang, Huijuan Chen. Critical revision of the manuscript for important intellectual content: Hao Zhang, Xuebing Chen.

(iii) All authors approved the final version of the manuscript.

**Conceptualization:** Xuebing Chen.

**Data curation:** Xuebing Chen, Hao Zhang, Shimei Ou, Huijuan Chen.

**Formal analysis:** Xuebing Chen, Hao Zhang, Shimei Ou, Huijuan Chen.

**Methodology:** Xuebing Chen, Hao Zhang, Shimei Ou, Huijuan Chen.

**Project administration:** Shimei Ou.

**Software:** Xuebing Chen, Hao Zhang.

**Writing – original draft:** Xuebing Chen, Hao Zhang.

**Writing – review & editing:** Xuebing Chen.

## Supplementary Material

Supplemental Digital Content

## References

[1] Ferlay J Soerjomataram I Dikshit R. Cancer incidence and mortality worldwide: sources, methods and major patterns in GLOBOCAN 2012. Int J Cancer 2015;136:E359–86. 25220842 10.1002/ijc.29210

[2] de Martel C Georges D Bray F Ferlay J Clifford GM. Global burden of cancer attributable to infections in 2018: a worldwide incidence analysis. Lancet Glob Health 2020;8:e180–90. 31862245 10.1016/S2214-109X(19)30488-7

[3] Liu J Yang HI Lee MH. Spontaneous seroclearance of hepatitis B seromarkers and subsequent risk of hepatocellular carcinoma. Gut 2014;63:1648–57. 24225939 10.1136/gutjnl-2013-305785

[4] Kao JH Chen PJ Lai MY Chen DS. Basal core promoter mutations of hepatitis B virus increase the risk of hepatocellular carcinoma in hepatitis B carriers. Gastroenterology 2003;124:327–34. 12557138 10.1053/gast.2003.50053

[5] Yang JH Zhang H Chen XB Chen G Wang X. Relationship between hepatocellular carcinoma and hepatitis B virus genotype with spontaneous YMDD mutations. World J Gastroenterol 2013;19:3861–5. 23840126 10.3748/wjg.v19.i24.3861 PMC3699046

[6] Yang J Chen X Zhang H Chen G. HBV genotype C strains with spontaneous YMDD mutations may be a risk factor for hepatocellular carcinoma. J Med Virol 2014;86:913–7. 24615989 10.1002/jmv.23895

[7] Tang H Oishi N Kaneko S Murakami S. Molecular functions and biological roles of hepatitis B virus x protein. Cancer Sci 2006;97:977–83. 16984372 10.1111/j.1349-7006.2006.00299.x PMC11159107

[8] Ng SA Lee C. Hepatitis B virus X gene and hepatocarcinogenesis. J Gastroenterol 2011;46:974–90. 21647825 10.1007/s00535-011-0415-9

[9] Dragani TA. Risk of HCC: genetic heterogeneity and complex genetics. J Hepatol 2010;52:252–7. 20022654 10.1016/j.jhep.2009.11.015

[10] Teufel A Marquardt JU Galle PR. Novel insights in the genetics of HCC recurrence and advances in transcriptomic data integration. J Hepatol 2012;56:279–81. 21782765 10.1016/j.jhep.2011.05.035

[11] Bartel DP. MicroRNAs: target recognition and regulatory functions. Cell 2009;136:215–33. 19167326 10.1016/j.cell.2009.01.002 PMC3794896

[12] Ambros V. The functions of animal microRNAs. Nature 2004;431:350–5. 15372042 10.1038/nature02871

[13] Bernig T Chanock SJ. Challenges of SNP genotyping and genetic variation: its future role in diagnosis and treatment of cancer. Expert Rev Mol Diagn 2006;6:319–31. 16706736 10.1586/14737159.6.3.319

[14] Bushati N Cohen SM. microRNA functions. Annu Rev Cell Dev Biol 2007;23:175–205. 17506695 10.1146/annurev.cellbio.23.090506.123406

[15] Lo Sardo F Pulito C Sacconi A. YAP/TAZ and EZH2 synergize to impair tumor suppressor activity of TGFBR2 in non-small cell lung cancer. Cancer Lett 2020. 10.1016/j.canlet.2020.11.037 33296708

[16] Lu J Zhu Y Williams S. Autism-associated miR-873 regulates ARID1B, SHANK3 and NRXN2 involved in neurodevelopment. Transl Psychiatry 2020;10:418. 33262327 10.1038/s41398-020-01106-8 PMC7708977

[17] Bandini E Fanini F Vannini I. miR-9-5p as a regulator of the androgen receptor pathway in breast cancer cell lines. Front Cell Dev Biol 2020;8:579160. 33282861 10.3389/fcell.2020.579160 PMC7689370

[18] Niu F Dzikiewicz-Krawczyk A Koerts J. MiR-378a-3p is critical for Burkitt lymphoma cell growth. Cancers (Basel) 2020;12:10.3390/cancers12123546 PMC7760147 33261009

[19] Chang HY Lee CH Li YS. MicroRNA-146a suppresses tumor malignancy via targeting vimentin in esophageal squamous cell carcinoma cells with lower fibronectin membrane assembly. J Biomed Sci 2020;27:102. 33248456 10.1186/s12929-020-00693-4 PMC7697386

[20] Wu C Tang G Wang X. Micro-RNA-21 rs1292037 A>G polymorphism can predict hepatocellular carcinoma prognosis (HCC), and plays a key role in cell proliferation and ischemia-reperfusion injury (IRI) in HCC cell model of IRI. Saudi Med J 2020;41:383–92. 32291425 10.15537/smj.2020.4.24994 PMC7841620

[21] Marangon CG de Bitencorte JT Michita RT. Association between MICA rs2596542 polymorphism with the risk of hepatocellular carcinoma in chronic hepatitis C patients. Pathol Oncol Res 2020;26:1519–25. 31471884 10.1007/s12253-019-00738-6

[22] Jiang ZC Tang XM Zhao YR Zheng L. A functional variant at miR-34a binding site in toll-like receptor 4 gene alters susceptibility to hepatocellular carcinoma in a Chinese Han population. Tumour Biol 2014;35:12345–52. 25179842 10.1007/s13277-014-2547-z

[23] Li Z Guo Y Zhou L. Association of a functional RAD52 genetic variant locating in a miRNA binding site with risk of HBV-related hepatocellular carcinoma. Mol Carcinog 2015;54:853–8. 24729511 10.1002/mc.22156

[24] Marrero JA Kulik LM Sirlin CB. Diagnosis, staging, and management of hepatocellular carcinoma: 2018 practice guidance by the American Association for the Study of Liver Diseases. Hepatology 2018;68:723–50. 29624699 10.1002/hep.29913

[25] Hou JL Wang G Wang F. The guideline of prevention and treatment for chronic hepatitis B: a 2015 update. Zhonghua Gan Zang Bing Za Zhi 2015;23:888–905. 26739464 10.3760/cma.j.issn.1007-3418.2015.12.002 PMC12677373

[26] Rehmsmeier M Steffen P Hochsmann M Giegerich R. Fast and effective prediction of microRNA/target duplexes. RNA 2004;10:1507–17. 15383676 10.1261/rna.5248604 PMC1370637

[27] Nicoloso MS Sun H Spizzo R. Single-nucleotide polymorphisms inside microRNA target sites influence tumor susceptibility. Cancer Res 2010;70:2789–98. 20332227 10.1158/0008-5472.CAN-09-3541 PMC2853025

[28] Pu F Shao Z Yang S. Association between functional variants in BIRC5/survivin gene 3′ untranslated region and mRNA expression in lymphoblastoid cell lines. Oncol Lett 2015;10:2319–22. 26622842 10.3892/ol.2015.3507 PMC4579978

[29] Zu Y Ban J Xia Z. Genetic variation in a miR-335 binding site in BIRC5 alters susceptibility to lung cancer in Chinese Han populations. Biochem Biophys Res Commun 2013;430:529–34. 23232114 10.1016/j.bbrc.2012.12.001

[30] Yang S Li JL Bi HC. Construction and function identification of luciferase reporter gene vectors containing SNPs in NFKBIA gene 3′UTR. Yao Xue Xue Bao 2016;51:80–5. 27405166

[31] Kim M Chen X Chin LJ. Extensive sequence variation in the 3′ untranslated region of the KRAS gene in lung and ovarian cancer cases. Cell Cycle 2014;13:1030–40. 24552817 10.4161/cc.27941 PMC3984301

[32] Li YH Wang##J Jiang F Lin WY Shen FM Meng W. Correlation of survivin gene with hepatocellular carcinoma in Han nationality in east China. Acad J Second Mil Med Univ 2010;31:1314–8.

[33] Gao J Xu HL Gao S. Genetic polymorphism of NFKB1 and NFKBIA genes and liver cancer risk: a nested case-control study in Shanghai, China. BMJ Open 2014;4:e004427. 10.1136/bmjopen-2013-004427 PMC3939648 24578542

[34] He Y Zhang H Yin J. IkappaBalpha gene promoter polymorphisms are associated with hepatocarcinogenesis in patients infected with hepatitis B virus genotype C. Carcinogenesis 2009;30:1916–22. 19797428 10.1093/carcin/bgp226 PMC2783005

[35] Xiong D Song YP Xiong W Liang YD. An let-7 KRAS rs712 polymorphism increases hepatocellular carcinoma risk. Genet Mol Res 2015;14:14050–5. 26535719 10.4238/2015.October.29.24

[36] McGlynn KA Petrick JL El-Serag HB. Epidemiology of hepatocellular carcinoma. Hepatology 2021;73: Suppl 1: 04–13. 10.1002/hep.31288 PMC7577946 32319693

[37] Sun J Jiang Z Li Y Wang K Chen X Liu G. Downregulation of miR-21 inhibits the malignant phenotype of pancreatic cancer cells by targeting VHL. Onco Targets Ther 2019;12:7215–26. 31564905 10.2147/OTT.S211535 PMC6732742

[38] Jonasch E Walker CL Rathmell WK. Clear cell renal cell carcinoma ontogeny and mechanisms of lethality. Nat Rev Nephrol 2021;17:245–61. 33144689 10.1038/s41581-020-00359-2 PMC8172121

[39] Sanford T Gomella PT Siddiqui R. Long term outcomes for patients with von Hippel-Lindau and Pheochromocytoma: defining the role of active surveillance. Urol Oncol 2020;39:134 e131–8. 10.1016/j.urolonc.2020.11.019 PMC9175510 33303379

[40] Yu F White SB Zhao Q Lee FS. HIF-1alpha binding to VHL is regulated by stimulus-sensitive proline hydroxylation. Proc Natl Acad Sci U S A 2001;98:9630–5. 11504942 10.1073/pnas.181341498 PMC55503

[41] Du J Zhang D Zhang W. pVHL negatively regulates antiviral signaling by targeting MAVS for proteasomal degradation. J Immunol 2015;195:1782–90. 26179906 10.4049/jimmunol.1500588

[42] Suenaga M Yamada S Fuchs BC. CD44 single nucleotide polymorphism and isoform switching may predict gastric cancer recurrence. J Surg Oncol 2015;112:622–8. 26416034 10.1002/jso.24056

[43] Na R Labbate C Yu H. Single-nucleotide polymorphism-based genetic risk score and patient age at prostate cancer diagnosis. JAMA Netw Open 2019;2:e1918145. 31880795 10.1001/jamanetworkopen.2019.18145 PMC6991229

[44] Bakshi D Nagpal A Sharma V. MassARRAY-based single nucleotide polymorphism analysis in breast cancer of north Indian population. BMC Cancer 2020;20:861. 32894086 10.1186/s12885-020-07361-8 PMC7487711

[45] Yang S Yu F Lin M. Single-nucleotide polymorphism rs17548629 in RIPK1 gene may be associated with lung cancer in a young and middle-aged Han Chinese population. Cancer Cell Int 2020;20:143. 32368189 10.1186/s12935-020-01215-w PMC7191703

[46] Bruix J Sherman M. Practice Guidelines Committee. American Association for the Study of Liver Diseases. Management of hepatocellular carcinoma. Hepatology 2005;42:1208–36. 16250051 10.1002/hep.20933

[47] Zoutendijk R Reijnders JG Zoulim F. Virological response to entecavir is associated with a better clinical outcome in chronic hepatitis B patients with cirrhosis. Gut 2013;62:760–5. 22490523 10.1136/gutjnl-2012-302024

[48] Bi J Zhang Z Qin E. Nucleoside analogs treatment delay the onset of hepatocellular carcinoma in patients with HBV-related cirrhosis. Oncotarget 2017;8:96725–31. 29228565 10.18632/oncotarget.18075 PMC5722517

[49] Fung J Cheung KS Wong DK. Long-term outcomes and predictive scores for hepatocellular carcinoma and hepatitis B surface antigen seroclearance after hepatitis B e-antigen seroclearance. Hepatology 2018;68:462–72. 29534307 10.1002/hep.29874

[50] Lo PH Urabe Y Kumar V. Identification of a functional variant in the MICA promoter which regulates MICA expression and increases HCV-related hepatocellular carcinoma risk. PLoS One 2013;8:e61279. 23593449 10.1371/journal.pone.0061279 PMC3623965

