# Supplementary material for: Von Hippel-Lindau gene single nucleotide polymorphism (rs1642742) may be related to the occurrence and metastasis of HBV-related hepatocellular carcinoma
Source: Medicine (Baltimore). 2021 Sep 3;100(35):e27187. doi: 10.1097/MD.0000000000027187 (PMC8415925; doi:10.1097/MD.0000000000027187)
Supplement: Supplemental Digital Content [file medi-100-e27187-s001.doc]

Supplement table 1 Signal pathways and genes interacting with HBxSignal pathway

|  | Gene symbol | | Full name |
| --- | --- | --- | --- |
| NF-kappa B signal pathway | RELA | | Transcription factor p65 |
| RELB | | v-rel avian reticuloendotheliosis viral oncogene homolog B | |
| REL | | Proto-oncogene c-Rel | |
| NFKB1 | | Nuclear factor NF-kappa-B p105 subunit | |
| NFKB2 | | Nuclear factor NF-kappa-B p100 subunit | |
| NFKBIA | | NFKB inhibitor alpha | |
| NFKBIB | | NF-kappa-B inhibitor beta | |
| NFKBIE | | NFKB inhibitor epsilon | |
| CHUK | | Inhibitor of nuclear factor kappa-B kinase subunit alpha | |
| BCL2L1 | | Bcl-2-like protein 1 | |
| JAK-STAT signal pathway | JAK1 | | Janus kinase 1 |
| JAK2 | | Janus kinase 2 | |
| JAK3 | | Janus kinase 3 | |
| TYK2 | | Tyrosine kinase 2 | |
| STAM | | Signal transducing adaptor molecule | |
| STAM2 | | Signal transducing adaptor molecule 2 | |
| STAT1 | | Signal transducer and activator of transcription 1 | |
| STAT2 | | Signal transducer and activator of transcription 2 | |
| STAT3 | | Signal transducer and activator of transcription 3 | |
| STAT4 | | Signal transducer and activator of transcription 4 | |
| STAT5A | | Signal transducer and activator of transcription 5A | |
| STAT5B | | Signal transducer and activator of transcription 5B | |
| STAT6 | | Signal transducer and activator of transcription 6 | |
| PTPN6 | | Protein tyrosine phosphatase, non-receptor type 6 | |
| PTPN11 | | Protein tyrosine phosphatase, non-receptor type 11 | |
| GRB2 | | Growth factor receptor bound protein 2 | |
| SOS1 | | Son of sevenless homolog 1 | |
| SOS2 | | Son of sevenless homolog 2 | |
| PIAS1 | | Protein inhibitor of activated STAT 1 | |
| PIAS2 | | Protein inhibitor of activated STAT 2 | |
| PIAS3 | | Protein inhibitor of activated STAT 3 | |
| PIAS4 | | Protein inhibitor of activated STAT 4 | |
| PI3K-Akt signal pathway | PIK3CA | | Phosphatidylinositol 4,5-bisphosphate 3-kinase catalytic subunit alpha isoform |
| PIK3CB | | Phosphatidylinositol 4,5-bisphosphate 3-kinase catalytic | |
